# Poly(3,3-dibenzyl-3,4-dihydro-2*H*-thieno[3,4-*b*][1,4]dioxepine)/Platinum Composite Films as Potential Counter Electrodes for Dye-Sensitized Solar Cells

**DOI:** 10.3390/polym9070271

**Published:** 2017-07-07

**Authors:** Jung-Chuan Chou, Yu-Chi Huang, Tzi-Yi Wu, Yi-Hung Liao, Chih-Hsien Lai, Chia-Ming Chu, Yu-Jen Lin

**Affiliations:** 1 Graduate School of Electronic Engineering, National Yunlin University of Science and Technology, Yunlin 64002, Taiwan; choujc@yuntech.edu.tw (J.-C.C.); chlai@yuntech.edu.tw (C.-H.L.); m10313328@gmail.com (Y.-J.L.); 2 Department of Electronic Engineering, National Yunlin University of Science and Technology, Yunlin 64002, Taiwan; B10113114@yuntech.edu.tw; 3 Graduate School of Chemical and Materials Engineering, National Yunlin University of Science and Technology, Yunlin 64002, Taiwan; M10415019@yuntech.org.tw; 4 Department of Information and Electronic Commerce Management, TransWorld University, Yunlin 64063, Taiwan; liaoih@twu.edu.tw

**Keywords:** dye-sensitized solar cells, electrochemical impedance spectroscopy, conducting polymer, photovoltaic conversion efficiency

## Abstract

In this study, poly(3,3-dibenzyl-3,4-dihydro-2*H*-thieno[3,4-*b*][1,4]dioxepine)/platinum composite films (PProDOT-Bz_2_/Pt) were used as counter electrodes (CEs) in dye-sensitized solar cells (DSSCs). The composite films were prepared on fluorine-doped tin oxide (FTO) glass by radio frequency (RF) sputtering to deposit platinum (Pt) for 30 s. Afterwards, PProDOT-Bz_2_ was deposited on the Pt–FTO glass via electrochemical polymerization. The electron transfer process of DSSCs was investigated using electrochemical impedance spectroscopy (EIS) and cyclic voltammetry (CV). The DSSCs with 0.05 C/cm^2^ PProDOT-Bz_2_-Pt composite films showed an open circuit voltage (*V*_oc_) of 0.70 V, a short-circuit current density (*J*_sc_) of 7.27 mA/cm^2^, and a fill factor (F.F.) of 68.74%. This corresponded to a photovoltaic conversion efficiency (η) of 3.50% under a light intensity of 100 mW/cm^2^.

## 1. Introduction

Dye-sensitized solar cells (DSSCs) are typical photoelectrochemical cells; their efficiencies are comparable to those of traditional p-n junction solar cells. DSSCs have attracted considerable interest due to their low cost of production, environmental friendliness, and low energy consumption [1,2,3,4,5,6,7,8]. The CE is an essential component of DSSCs; it injects electrons into the electrolyte to catalyze the reduction reaction from triiodide ion to iodide ion after a charge injection from the dye [9]. In this regard, Pt has excellent electrical conductivity and high catalytic activity, making it suitable for use as a CE. However, the platinum electrode suffers several challenges: (i) Platinum is expensive and the quantity of platinum worldwide is too small to supply the increasing demands for several fields involving catalysis; (ii) platinum electrodes are easily eroded by iodine-based electrolytes, which will create by-products like PtI_4_ and H_2_PtI_6_ in liquid-state DSSCs; (iii) Pt as a CE in DSSCs is not effective for the I-free redox couple (such as Co^3+^/Co^2+^ and T_2_/T^−^) electrolyte; (iv) Pt electrode can be poisoned by sulfur or phosphate species; (v) the platinum electrode cannot match the novel materials of working electrodes, novel dyes, and novel electrolytes in emerging solar cells. These shortcomings of Pt influence CE stability significantly and give rise to degenerated solar cell performance [10,11]. Conducting polymers (CPs) are promising CEs due to their ease of processing from a solution, CPs have excellent catalytic activity in the metallic state and show great potential to produce large-area and low-cost CEs. In recent years, polyaniline (PANI) [12,13], polypyrrole (PPy) [14], poly(3,4-ethylenedioxythiophene) (PEDOT) [15,16,17,18], poly(3,4-ethylenedioxythiophene):poly(4-styrenesulfonate) (PEDOT:PSS) [19,20,21], polythiophene (PTh) [10], and poly(3,3-diethyl-3,4-dihydro-2*H*-thieno-[3,4-*b*][1,4]-dioxepine) (PProDOT-Et_2_) [21,22] have been reported as potential CEs of DSSCs. Moreover, DSSCs using CP-based electrolytes have many benefits associated with solvent-free devices such as improved long-term stability, high thermal stability, low liquid leakage, and so forth [23,24,25,26,27,28,29,30]. However, the applications of poly(3,3-dibenzyl-3,4-dihydro-2*H*-thieno[3,4-*b*][1,4]dioxepine) (PProDOT-Bz_2_) in CE of DSSCs are not reported so far. In the present work, cell performances of the DSSCs assembled based on the prepared PProDOT-Bz_2_/Pt composite films as CEs were characterized. The authors anticipate that this work will stimulate further scientific research and finally be beneficial in selecting suitable CEs to improve the performance of DSSCs in the future.

## 2. Materials and Methods

### 2.1. Materials

Titanium dioxide (TiO_2_) powder (P25) was purchased from Degussa, Essen, Germany. The P25 was 80% anatase and 20% rutile. The graphene oxide (GO) was purchased from Tokyo Chemical Industry Co., Ltd., Tokyo, Japan. Ruthenium-535 (N3) was purchased from Solaronix, Aubonne, Switzerland. Absolute ethanol was purchased from Katayama Chemical, Osaka, Japan. Acetylacetone (AcAc) was purchased from Acros Organics, Geel, Belgium. Lithium iodide (LiI) and 4-*tert*-butylpyridine (TBP) were purchased from Sigma-Aldrich, St. Louis, MO, USA. Iodine (I_2_) was purchased from Riedel-deHaen, Seelze, Germany. 1-propyl-2,3-dimethylimidazolium iodide (DMPII) was purchased from Tokyo Chemical Industry Co., Ltd., Tokyo, Japan. Fluorine-doped tin oxide (FTO) glass substrate was purchased from C.P. Solar, Co., Ltd., Kaohsiung, Taiwan. 3,3-dibenzyl-3,4-dihydro-2*H*-thieno[3,4-*b*][1,4]dioxepine (ProDOT-Bz_2_) was synthesized using our previous procedures [31,32,33].

### 2.2. Fabrication of PProDOT-Bz_2_ Counter Electrodes

The various Pt–FTO glass substrates were prepared by using radio frequency (RF) sputtering system and sputtering Pt for 30 and 360 s onto FTO glass, respectively. The thickness of Pt layer sputtering for 360 s is 90 nm. The sputtering parameters of Ar flow rate, working pressure, and radio frequency sputtering power were kept at 10 sccm, 30 mTorr, and 60 W, respectively. PProDOT-Bz_2_/Pt films were prepared by electrochemical polymerization. The electrochemical system comprised 30 s Pt–FTO glass as a working electrode, Pt wire as a counter electrode, and Ag/AgCl as a reference electrode. PProDOT-Bz_2_ was synthesized on 30 s Pt–FTO glass using 0.1 M LiClO_4_, 0.015 M ProDOT-Bz_2_ monomer and acetonitrile as a solvent. The polymerized potential was set at 1.55 V until the charge capacities were reached 25, 50, and 100 mC, respectively. The prepared PProDOT-Bz_2_/30 s films were dipped in acetonitrile to wash out the unreacted ProDOT-Bz_2_ monomer and were kept in an oven at 60 °C for 1 h.

### 2.3. Assembly of the DSSCs

**Figure 1:**
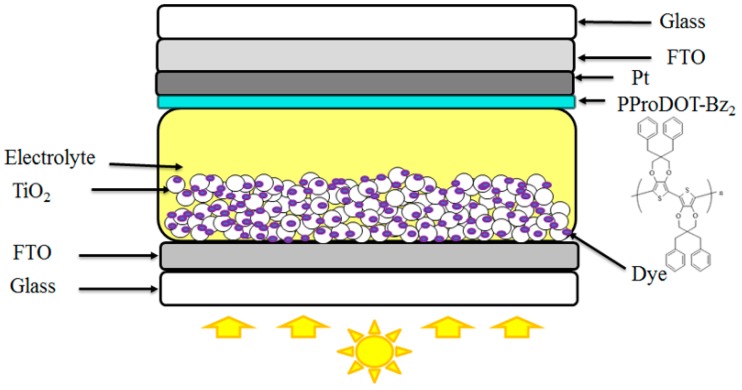
The structure of dye-sensitized solar cells.

The FTO glass was cleaned by sonicating it in acetone, ethanol, and deionized water for 10 min, respectively. 3 g titanium dioxide (TiO_2_) powder, 4 mL deionized water, 0.05 mL acetylacetone (AcAc), 0.15 mL Triton X-100, and 2 mL graphene oxide (GO) were placed into bottles and stirred for 12 h. TiO_2_ colloid was deposited on the FTO glass via the spin coating method at 1000 rpm for 11.5 s followed by coating at 2500 rpm for 15 s and was sintered at 450 °C in an annealing furnace for 30 min. Afterwards, a TiO_2_ mixture consisting of 0.25 g TiO_2_ powder, 0.025 g iodine (I_2_), and 25 mL AcAc was coated on the top of the TiO_2_/GO layer by electrophoretic deposition [34]. Finally, the double-layer TiO_2_ photoelectrode was annealed at 450 °C for 30 min again. The prepared film with an active area of 0.25 cm^2^ was immersed in ethanol solution containing 0.5 mM N3 dye. The DSSC electrolyte consisted of 0.6 M DMPII, 0.5 M LiI, 0.05 M I_2_, and 0.5 M TBP in 15 mL MPN. The as-prepared TiO_2_ photoelectrode and CE were separated by a 65 μm thick Teflon tape filled with an electrolyte. The structure of dye-sensitized solar cells is shown in Figure 1.

### 2.4. Characterization

The photovoltaic performances of DSSCs were measured using a solar simulator (MFS-PV-Basic-HMT, Shulin, Taiwan) and the intensity of incident sunlight was 100 mW/cm^2^. The Nyquist plot and electrochemical behavior of DSSC were investigated by electrochemical impedance spectroscopy (EIS, BioLogic SP-150, Seyssinet-Pariset, France), which were measured in the dark under a bias of −0.7 V. The frequency of EIS was set from 1 MHz to 50 mHz and an AC perturbation signal was set at 10 mV. The morphologies of CEs were observed by scanning electron microscope (SEM, JEOL JSM-7800F, Tokyo, Japan). Cyclic voltammetry measurements were implemented using a CHI660a electrochemical analyzer (CH Instruments, Austin, TX, USA).

## 3. Results and Discussion

### 3.1. Surface Morphology of the PProDOT-Bz_2_ Film

**Figure 2:**
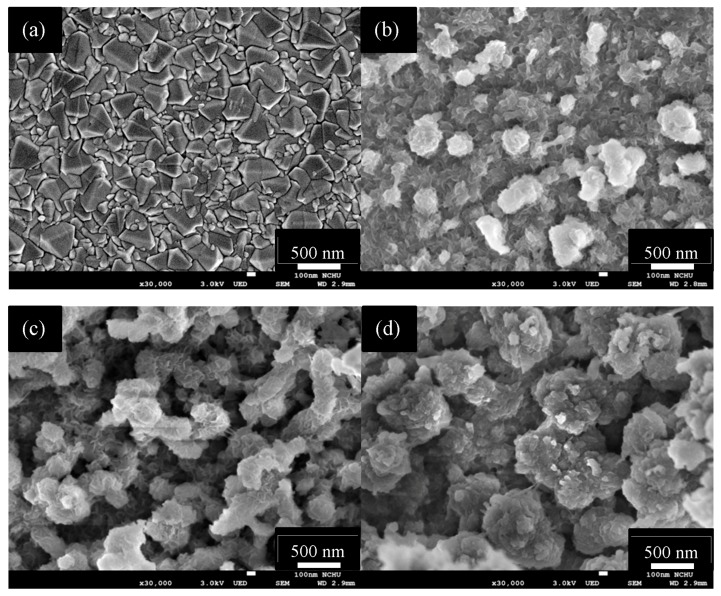
SEM images of the (**a**) 30 s Pt–FTO; (**b**) 0.025 C/cm^2^ PProDOT-Bz_2_/30 s Pt–FTO; (**c**) 0.05 C/cm^2^ PProDOT-Bz_2_/30 s Pt–FTO; (**d**) 0.1 C/cm^2^ PProDOT-Bz_2_/30 s Pt–FTO.

The surface morphology of PProDOT-Bz_2_/Pt composite films was studied using SEM images, as shown in Figure 2. Figure 2a shows the surface morphology of 30 s Pt–FTO glass; the surface morphologies of PProDOT-Bz_2_ with 0.025, 0.05, and 0.1 charge capacity (0.025, 0.05, and 0.1 C/cm^2^) polymerized on 30 s Pt–FTO glass are shown in Figure 2b–d, respectively. The SEM images indicate that the PProDOT-Bz_2_/30 s Pt composite films exhibited a three-dimensional porous network structure, which means electroactive sites may have increased in the composite films for I_3_^−^ to I^−^. The porous morphology of PProDOT-Bz_2_ film could facilitate the penetration of DSSC electrolyte, and the charge transfer resistance decreased at the CE/electrolyte interface [6]. The resultant porous structure of the PProDOT-Bz_2_/30 s Pt composite films could also facilitate the penetration of the electrolyte in CEs of DSSCs, resulting in a reduced charge transfer resistance at the CE/electrolyte interface. However, the porous structure of the PProDOT-Bz_2_/30 s Pt composite films decreased with the increasing charge capacity until 0.1 C, a proper electron transfer process could not be expected from the substrate to the composite film and to the triiodide ions in DSSC electrolyte. Accordingly, the charge transfer efficiency of PProDOT-Bz_2_/30 s Pt composite film decreased [35].

### 3.2. Photovoltaic Performances

**Figure 3:**
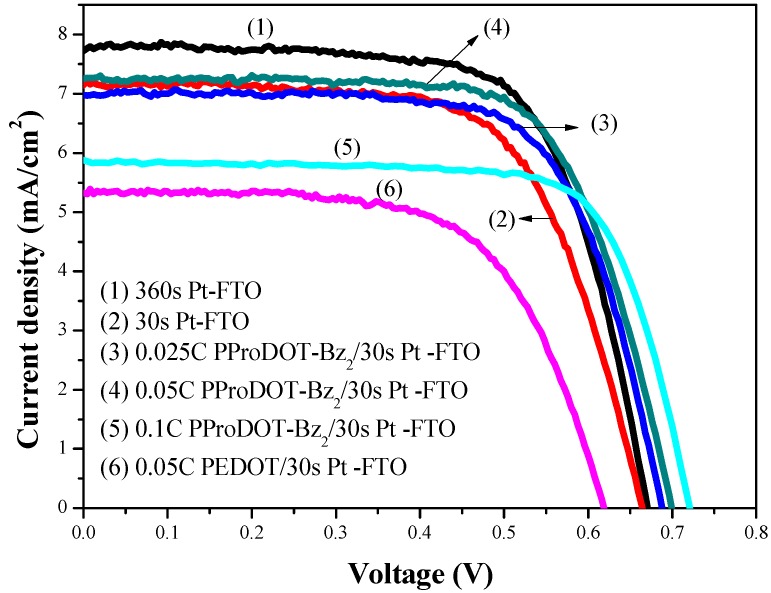
*J–V* curves of the DSSCs based on 360 s Pt, 30 s Pt, 0.025 C/cm^2^ PProDOT-Bz_2_/30 s Pt, 0.05 C/cm^2^ PProDOT-Bz_2_/30 s Pt, and 0.1 C/cm^2^ PProDOT-Bz_2_/30 s Pt counter electrodes, respectively. The DSSC electrolyte is based on 0.6 M DMPII, 0.5 M LiI, 0.05 M I_2_, and 0.5 M TBP in 15 mL MPN.

Figure 3 shows the current density–voltage (*J–V*) curves of the DSSCs based on various CEs under AM 1.5 G (100 mW/cm^2^) light illumination. The photovoltaic parameters such as the short-circuit current density (*J*_sc_), open circuit voltage (*V*_oc_), fill factor (F.F.) and photovoltaic conversion efficiency (η) are summarized in Table 1. The DSSC with sputtering time of 30 s Pt–FTO film showed lower efficiency (3.11%) than that sputtered for 360 s Pt–FTO film (3.58%). For the DSSCs fabricated with PProDOT-Bz_2_/Pt composite films, the photovoltaic conversion efficiency increased from 3.33% to 3.50% as the charge capacity increased from 0.025 to 0.05 C/cm^2^. As the charge capacity increased to 0.1 C/cm^2^, the photovoltaic conversion efficiency decreased to 2.08%. 0.1 C/cm^2^ PProDOT-Bz_2_/30 s Pt CE-based DSSC showed lower photovoltaic conversion efficiency value than those of other CEs, this can be attributed to 0.1 C/cm^2^ PProDOT-Bz_2_/30 s Pt CE showing lower catalytic activity and lower surface area than those of other CEs, as confirmed by the SEM images in Figure 2. In addition, we also compared the photovoltaic performances of 0.05 C/cm^2^ PProDOT-Bz_2_/30 s Pt CE-based DSSC with 0.05 C/cm^2^ PEDOT/30 s Pt CE-based DSSC. 0.05 C/cm^2^ PProDOT-Bz_2_/30 s Pt CE-based DSSC displays higher photovoltaic conversion efficiency value than that of 0.05 C/cm^2^ PEDOT/30 s Pt CE-based DSSC.

### 3.3. Electrochemical Impedance Spectra

**Figure 4:**
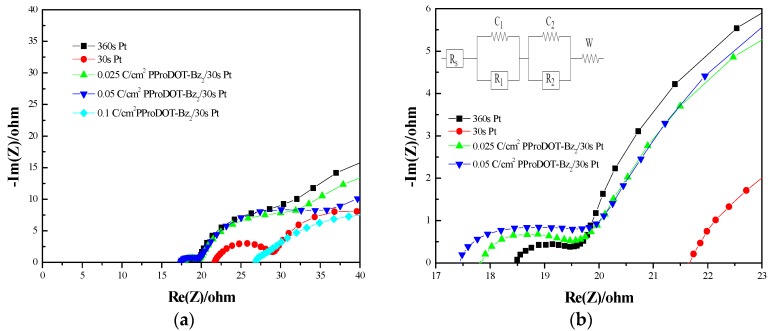
(**a**) Nyquist plots of the DSSCs based on 360 s Pt, 30 s Pt, 0.025 C/cm^2^ PProDOT-Bz_2_/30 s Pt, 0.05 C/cm^2^ PProDOT-Bz_2_/30 s Pt, and 0.1 C/cm^2^ PProDOT-Bz_2_/30 s Pt counter electrodes; (**b**) an enlarged graph of (**a**).

EIS analysis was employed to study the charge transfer resistances at the interface of the electrode. The Nyquist plots of the DSSC with different CEs in the measured frequency range from 1 to 50 MHz are shown in Figure 4, and their corresponding parameters are listed in Table 2. The *R*_s_ in the equivalent circuit corresponds to the ohmic series resistance of the substrate and its catalytic layer. The value could be evaluated by using the onset point of the first semicircle (left-hand side) in the high frequency region. A lower *R*_s_ refers to a better attachment of the catalytic film onto the substrate and therefore to a better conductivity of the film and better fill factor (F.F.) of the pertinent DSSC [18]. The first semicircle in the high-frequency zone of impedance spectra denotes the charge transfer resistance at CE/electrolyte interface (*R*_1_), implying the catalytic properties of the CEs towards triiodide ion reduction [10]. A lower *R*_1_ can be attributed to higher electrocatalytic activity from the reduction of I_3_^−^ to I^−^. The second semicircle in the middle-frequency region is related to the resistance at the TiO_2_/dye/electrolyte (*R*_2_). The third semicircle in the low-frequency region denotes the Warburg diffusion resistance of the electrolyte (*W*) [15]. DSSCs are fabricated using various CEs, the resistance between the CE and electrolyte (*R*_1_) is a crucial parameter for the influence of various CEs. A lower R_1_ can be ascribed to a higher electrocatalytic activity from the reduction of I_3_^−^ to I^−^ at CE/electrolyte interface. 0.025 C/cm^2^ PProDOT-Bz_2_/30 s Pt–FTO and 0.05 C/cm^2^ PProDOT-Bz_2_/30 s Pt–FTO show lower *R*_1_ values than that of 30 s Pt–FTO. These results may be attributed to the high conductivity and porous structure of these PProDOT-Bz_2_/30 s Pt films that increased the electron transfer and facilitated the penetration of electrolyte in CEs of DSSCs [13].

### 3.4. Cyclic Voltammetry

**Figure 5:**
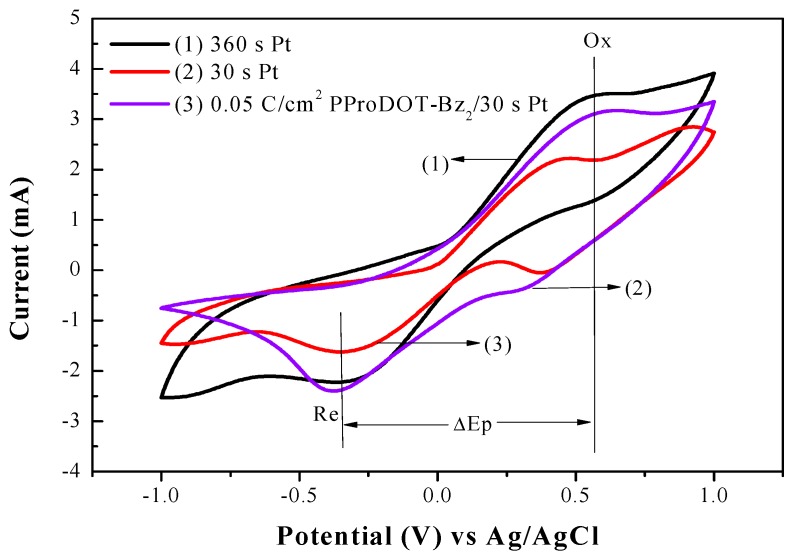
Cyclic voltammetry of the various counter electrodes at a scan rate of 100 mV s^−1^ in acetonitrile solution containing 0.1 M LiClO_4_, 10 mM LiI, and 1 mM I_2_.

The electrochemical measurement of cyclic voltammetry (CV) was carried out using three electrode systems; various CEs were used as working electrodes, a platinum wire was used as a counter electrode, and Ag/AgCl was used as a reference electrode. The CV measurements were used to evaluate the electrocatalytic activities of 360 s Pt, 30 s Pt, and 0.05 C PProDOT-Bz_2_/30 s Pt CEs in acetonitrile solution containing 0.1 M LiClO_4_, 10 mM LiI, and 1 mM I_2_. In general, the electrocatalytic activity of a CE is determined using two parameters, one is the separation of anodic and cathodic peak (Δ*E*_p_), and the other is cathodic peak current density (*I*_pc_). The overall electrocatalytic ability of CEs was characterized using these parameters. A larger *I*_pc_ denotes greater electrocatalytic ability, and a smaller Δ*E*_p_ indicates a lower overpotential toward the catalytic reduction of the triiodide ion to the iodide ion in DSSCs [10,25]. Compared with 30 s Pt CE as shown in Figure 5, 0.05 C PProDOT-Bz_2_/30 s Pt CE exhibits a higher *I*_pc_ than that of 30 s Pt CE. In addition, these CEs show similar Δ*E*_p_, indicating similar overpotentials toward the catalytic reduction of the triiodide ion to the iodide ion. 0.05 C PProDOT-Bz_2_/30 s Pt CEs exhibits higher overall electrocatalytic ability than that of 30 s Pt CE, this may be attributed to 0.05 C PProDOT-Bz_2_/30 s Pt composite films possess porous structure. High active area of porous structure facilitates the reduction reaction of I^−^/I_3_^−^.

## 4. Conclusions

Poly(3,3-dibenzyl-3,4-dihydro-2*H*-thieno[3,4-*b*][1,4]dioxepine)/platinum composite films (PProDOT-Bz_2_/Pt) were used as the CEs in DSSCs. The DSSC based on 0.05 C PProDOT-Bz_2_/30 s Pt composite CE exhibited a photovoltaic conversion efficiency of 3.50%, which was higher than that with 30 s Pt CE (3.11%) and up to 98% of that with 360 s Pt CE (3.58%) under the 100 mW/cm^2^ light illumination. The results indicate that 0.05 C PProDOT-Bz_2_/30 s Pt composite films represent a promising substitute for the expensive Pt as CE is for DSSC.

## Figures and Tables

**Table 1:** The photovoltaic parameters of DSSCs with various counter electrodes.

Counter Electrode	*V*_oc_ (V)	*J*_sc_ (mA/cm^2^)	F.F. (%)	η (%)
360 s Pt	0.67 ± 0.02	7.80 ± 0.25	68.61 ± 2.77	3.58 ± 0.10
30 s Pt	0.67 ± 0.03	7.20 ± 0.31	64.94 ± 2.34	3.11 ± 0.11
0.025 C/cm^2^ PProDOT-Bz_2_/30 s Pt	0.69 ± 0.02	7.03 ± 0.45	68.82 ± 2.90	3.33 ± 0.11
0.05 C/cm^2^ PProDOT-Bz_2_/30 s Pt	0.70 ± 0.04	7.27 ± 0.35	68.74 ± 2.25	3.50 ± 0.13
0.1 C/cm^2^ PProDOT-Bz_2_/30 s Pt	0.62 ± 0.03	5.31 ± 0.33	64.46 ± 2.83	2.08 ± 0.13
0.05 C/cm^2^ PEDOT/30 s Pt	0.72 ± 0.04	5.88 ± 0.28	73.09 ± 2.14	3.10 ± 0.14

**Table 2:** The resistance and capacitance of equivalent circuit for DSSCs.

Counter Electrode	*R_s_* (Ω)	*C*_1_ (µF)	*R*_1_(Ω)	*C*_2_ (mF)	*R*_2_ (Ω)
360 s Pt–FTO	18.42	85.63	1.05	5.12	12.57
30 s Pt–FTO	21.65	27.49	7.40	3.61	15.71
0.025 C/cm^2^ PProDOT-Bz_2_/30 s Pt–FTO	17.82	38.05	1.65	3.89	12.43
0.05 C/cm^2^ PProDOT-Bz_2_/30 s Pt–FTO	17.43	66.63	2.08	3.49	10.57
0.1 C/cm^2^ PProDOT-Bz_2_/30 s Pt–FTO	26.85	0.013	15.14	3.04	51.47
